# Effect of Pre-Treating Dietary Green Seaweed with Proteolytic and Fibrolytic Enzymes on Physiological and Meat Quality Parameters of Broiler Chickens

**DOI:** 10.3390/foods10081862

**Published:** 2021-08-12

**Authors:** Tumisang Ben Matshogo, Caven Mguvane Mnisi, Victor Mlambo

**Affiliations:** 1 Department of Animal Science, School of Agricultural Science, North-West University, P Bag x2046, Mafikeng 2745, South Africa; matshogo1@gmail.com; 2 Food Security and Safety Niche Area, Faculty of Natural and Agricultural Science, North-West University, P Bag x2046, Mafikeng 2745, South Africa; 3 Faculty of Agriculture and Natural Sciences, School of Agricultural Sciences, University of Mpumalanga, P Bag x11283, Nelspruit 1200, South Africa; victor.mlambo@ump.ac.za

**Keywords:** blood parameters, chickens, growth performance, carcass characteristics, meat quality

## Abstract

The use of seaweeds as nutraceuticals in chicken diets is limited by high fibre levels and low protein digestibility. Therefore, we tested the effect of pre-treating dietary seaweed (*Ulva* sp.) with a combination of protease and fibrolytic enzymes on physiological and meat quality parameters of Cobb 500 broilers. Five dietary treatments were formulated by including untreated (T1); fibrolytic (12 g/kg) enzyme-treated (T2); fibrolytic (12 g/kg) and protease (5 g/kg) enzyme-treated (T3); fibrolytic (12 g/kg) and protease (10 g/kg) enzyme-treated (T4); fibrolytic (12 g/kg) and protease (15 g/kg) enzyme-treated (T5) seaweed (35 g/kg) in a standard broiler diet. Three hundred, two-week-old chicks (239.3 ± 8.57 g live weight) were evenly distributed to 30 replicate pens to which the diets were then randomly allocated. Birds fed diet T1 had the highest feed intake (1144.5 g/bird). Neither linear nor quadratic trends were recorded for growth performance and carcass traits in response to protease pre-treatment levels. Gizzard weight linearly increased, while symmetric dimethylarginine, calcium, meat pH_24_, and hue angle_24_ quadratically responded to protease levels. Diet T1 promoted the lowest serum phosphorus levels (3.37 mmol/L). In conclusion, pre-treatment of seaweed with a combination of protease and fibrolytic enzymes did not improve diet utilization, physiological parameters, and meat quality in broilers.

## 1. Introduction

The poultry industry significantly contributes to agri-food value chains around the world [1]. Chicken meat production has been increasing worldwide [2], driven by a growing human population, which is projected to breach the 9 billion mark by 2050 [3]. Over the years, poultry producers have relied heavily on high-performing conventional chicken strains to meet the increasing demand for animal protein. However, these chicken strains have high nutrient requirements resulting in high feeding costs and lower profit margins. In addition, intensive production systems predispose these chickens to a variety of stressors leading to a high incidence of diseases. Consequently, antibiotic growth promoters (AGP) have traditionally been used to control infections and enhance feed utilization efficiency [4]. However, the emergence of antimicrobial resistance as well as the threats posed by antibiotic residues to consumer health has seen AGPs being banned in some countries [4,5]. However, due to a lack of effective alternatives, the use of AGPs has continued in low-income countries that are burdened by high incidences of infectious disease outbreaks. A possible solution is the use of readily available plant-based feed additives with nutraceutical properties as AGP alternatives in poultry production.

Seaweeds, also known as marine macroalgae, are important sources of bioactive compounds that are not available in terrestrial plants [6]. These compounds that include polyphenols, pigments (chlorophyll, fucoxanthin, and phycobilins), and essential polyunsaturated fatty acids, have putative antioxidative, prebiotic, anti-inflammatory, immunomodulatory, antimicrobial, and cholesterol lowering effects [7,8]. Apart from their multiple uses in food, cosmetic, medicine, and industrial sectors, seaweeds have contributed greatly to improved nutritional status of humans due to its rich composition of essential nutrients [9]. In addition, seaweeds have been utilized in animal feeds as rich sources of carbohydrates, protein, essential amino acids, minerals, and vitamins [10,11]. Evans and Critchley [10] reported that seaweeds have prebiotic properties that have health benefits to chickens. The inclusion of seaweeds in poultry feeds has the potential to improve feed utilization efficiency, bird health, and product quality [12]. However, seaweeds contain high levels of cellulose, ulvan, pectin, and xylan [8] that may reduce its utility for broilers. This is because broilers have a limited capacity to digest fibre resulting in impaired utilization of nutrients and weight gain [13]. Indeed, high levels of fibre reduce digestibility of nutrients in chickens [14]. Indeed, a strong negative correlation has been reported between fibre and protein digestibility in poultry [13]. In addition, the presence of condensed tannins in seaweeds could interfere with the utilization of proteins and further reduce its digestibility [14]. In our previous study [12], we found that feed utilization efficiency and growth performance were compromised when seaweed inclusion levels exceeded 35 g/kg in broiler diets. Higher seaweed inclusion levels in broiler diets would contribute to a more sustainable enterprise but requires the amelioration of fibre and enhancement of protein bioavailability.

Pre-treatment of feeds offered to birds with enzymes improves nutrient utilization and growth performance, lowers feed costs, and reduces environmental pollution [15,16]. Several enzyme preparations have also been used to extract polysaccharides, proteins, or phenolics from seaweeds [17]. For example, Yaich et al. [18] obtained sulfated polysaccharides with antioxidant properties from green seaweed (*Ulva lactuca*) after performing an enzymatic treatment with cellulases and proteases. However, the synergistic effects of a proteolytic mono-enzyme and fibrolytic multi-enzymes have not been explored for seaweeds used in broiler diets. If successful, this strategy could enable the dietary inclusion of higher levels of seaweeds resulting in more bioactive compounds and nutrients being available to the birds. Accordingly, the current study evaluated how pre-treating dietary green seaweed (*Ulva* sp.) with a combination of a protease mono-enzyme and fibrolytic multi-enzyme mixture affects the growth performance, hemato-biochemical parameters, internal organs, and carcass and meat quality characteristics in Cobb 500 broilers. It was hypothesized that enzymatic pre-treatment of dietary green seaweed would improve the physiological parameters and meat quality traits of the chickens.

## 2. Materials and Methods

### 2.1. Study Site and Ingredient Sources

The feeding trial was conducted at the North-West University Experimental Farm (26°41′36″ S, 27°05′35″ E) in South Africa from October to November 2020. The green seaweed (*Ulva sp.*) was harvested as described by Nhlane et al. [11] from an abalone farm (Western Cape, South Africa). The seaweed was oven-dried (60 °C) then milled (2 mm, Retsch grinder, 42781 Haan, Germany) to produce seaweed powder (SWP). The SWP was chemically analyzed prior to diet formulation and the chemical composition is reported in Nhlane et al. [11]. The fibrolytic enzyme mixture (Viscozyme^®^ L) was purchased from Sigma-Aldrich (Modderfontein, South Africa), and is composed of cellulase, hemicellulose, xylanase, *β*-glucanase, and arabanase enzymes. The Viscozyme^®^ L is completely miscible with water and has an enzyme activity of 100 fungal beta-glucanase per gram and a density of 1.2 g/mL. The protease mono-enzyme, as well as the other feed ingredients, were purchased from Nutroteq (PTY) LTD (Pretoria, South Africa). The protease has an enzyme activity of 600,000 U/g.

### 2.2. Feed Formulation and Analysis

Five dietary treatments were formulated (Table 1) to be isonitrogenous and isoenergetic by including SWP in a standard grower or finisher diet based on feed formulae provided by Nutroteq (PTY) LTD (Gauteng, South Africa). The experimental diets, in mash form, were formulated by including untreated (T1); fibrolytic (12 g/kg) enzyme-treated (T2); fibrolytic (12 g/kg) and protease (5 g/kg) enzyme-treated (T3); fibrolytic (12 g/kg) and protease (10 g/kg) enzyme-treated (T4); fibrolytic (12 g/kg) and protease (15 g/kg) enzyme-treated (T5) seaweed (35 g/kg) in a standard broiler diet. The inclusion levels of the SWP (35 g/kg) and the fibrolytic enzyme (12 g/kg) were based on findings from our previous study [12]. The nutritional composition (Table 2) of the untreated seaweed diet was analyzed as reported by Matshogo et al. [12].

### 2.3. Feeding Trial and Broiler Management

Three hundred Cobb 500 chicks (male, one day old) were bought from Super Birds farm (North West, South Africa). The chicks were weighed and randomly placed in 30 replicate pens (experimental units) holding 10 birds each. The pens measured 3.5 m × 1.0 m × 1.85 m (L × B × H) with sunflower husks as bedding. The chicks were given a stress pack for the first 3 days and reared on a commercial starter mash diet until day 10 of age. At day 11, the birds were adapted to the experimental diets until day 13 of age. The house temperature was maintained at 35 °C using infrared electric bulbs placed in the brooder to ensure constant supply of heat for the first two weeks. Measurements commenced from day 14 to day 28 for the grower phase and from day 29 to day 42 for the finisher phase. The birds had unlimited access to clean fresh water and feed for the entire duration of the feeding trial.

Amount of feed consumed was determined every morning by subtracting the amount of refused feed from the amount offered. The initial body weights (239.3 ± 8.57 g live weight) of the birds were measured at 2 weeks of the age. Thereafter, the birds were weighed weekly to determine average weekly weight gain (ABWG). The ratio of weight gain to feed consumption was used as a measure of feed conversion efficiency (FCE).

### 2.4. Blood Collection and Analysis

At day 40 of age, 4 mL of blood samples were collected early in the morning before feeding from 12 birds randomly selected per dietary treatment. The blood samples were collected from the branchial vein using 23 *gauge* disposable needles and 5 mL syringes. After each collection, the samples (2 mL) were immediately transferred into labelled serum and whole blood tubes. The automated LaserCyte Hematology and the Vet Test Chemistry Analyzer machines (IDEXX Laboratories SA (PTY) LTD, Gauteng, South Africa) were used to determine hematological and serum biochemical parameters, respectively.

### 2.5. Slaughter, Internal Organs, and Carcass and Meat Quality Traits

At day 42 of age, feed was withheld for 13 h before the birds were weighed to determine final body weight (FBW). The birds were then taken to a commercial abattoir (Rooigrond, North West, South Africa) where they were electrically stunned and slaughtered by cutting the jugular vein [12]. After bleeding, the carcasses were plucked and eviscerated to determine visceral organ weights (liver, gizzard, proventriculus, spleen, duodenum, jejunum, ileum, and caecum), carcass weights and carcass cuts (wing, breast, drumstick, and thigh), as well as meat quality parameters as previously described by Kumanda et al. [14].

Breast meat pH and color coordinates (L* = lightness, a* = redness and b* = yellowness) were measured as described by Matshogo et al. [12] and Kumanda et al. [14]. The color coordinates a* and b* were used to calculate hue angle and chroma values [19]. Water-binding capacity (WBC) of breast meat samples was determined following the filter-paper press method by Grau & Hamm [20]. Breast meat drip loss and cooking loss were determined as described by Honikel et al. [21]. Shear force values (N) for raw breast meat samples were determined [22] as described by Matshogo et al. [12].

### 2.6. Statistical Analysis

Data were first tested for normality using the NORMAL option in the Procedure Univariate statement and for homogeneity of variances using Levene’s test. Except for T1 data, linear and quadratic coefficients for physiological and meat quality data were evaluated using response surface regression analysis [23]. Weekly measured data were analyzed using the repeated measures analysis option in the general linear models (GLM) procedure of SAS version 9.4 [23] to determine the interactive effect of time (in weeks) and diet. Overall growth performance, hemato-biochemical parameters, and meat quality data were analyzed using one-way ANOVA (PROC GLM; [23]), where diet was the only variable. Significance was considered at *p* < 0.05 for all statistical tests and the least squares means were compared by using the probability of difference option in SAS.

## 3. Results

### 3.1. Growth Performance and Hemato-Biochemical Parameters

Repeated measures analysis indicated that there were no significant week × diet interaction effects on average weekly body weight gain (*p* = 0.946) and average weekly FCE (*p* = 0.124). However, an interaction effect was observed on average weekly feed intake (*p* = 0.018). Table 3 shows that there were no significant linear and quadratic effects for average weekly feed intake. However, significant dietary effects were observed in week 6 of age, where birds fed diet T1 had a higher (*p* < 0.05) feed intake (1144.5 g/bird) than those fed diets T2, T3, and T5, whose feed intake values did not differ.

Neither linear nor quadratic trends (*p* > 0.05) were recorded for overall body weight gain (BWG), overall FCE, or final body weight (FBW) in response to proteolytic enzyme treatment levels (Table 4). Similarly, no significant dietary influences were observed on overall BWG, overall FCE, or FBW of Cobb 500 broiler chickens.

Table 5 shows that there were neither linear nor quadratic responses for hemato-biochemical parameters, except for symmetric dimethylarginine (SDMA) (*y* = 10.71 (±1.759) + 1.23 (±0.565) *x* − 0.098 (± 0.036) *x*^2^; R^2^ = 0.241, *p* = 0.013) and calcium (*y* = 1.03 (±0.139) + 0.148 (±0.045) *x* − 0.01 (±0.003) *x*^2^; R^2^ = 0.350, *p* = 0.005) which quadratically responded to protease treatment levels. Dietary treatments only influenced (*p* > 0.05) SDMA, calcium, and phosphorus levels of the chickens. Birds fed diet T3 (16.0 µg/dL) had higher (*p* < 0.05) SMDA levels than those fed with diet T5 (7.50 µg/dL). The untreated seaweed control diet (T1) promoted the lowest phosphorus levels (3.37 mmol/L) compared to all the other dietary treatments, whose phosphorus levels did not differ (*p* > 0.05). Diet T2 promoted the lowest (*p* < 0.05) calcium content (1.03 mmol/L) compared to diets T3 (1.51 mmol/L) and T4 (1.57 mmol/L).

### 3.2. Carcass Characteristics, Internal Organs, and Meat Quality

Neither linear nor quadratic trends were observed for carcass characteristics and internal organs, except for gizzard weight, which linearly increased (*y* = 0.055 (±0.022) *x* + 1.88 (±0.069); R^2^ = 0.201, *p* = 0.022) as protease pre-treatment levels increased (Table 6). Likewise, dietary treatments had no influence (*p* > 0.05) on carcass traits and internal organ weights except for gizzard weights, where birds on treatments T3, T4, and T5 had the highest (*p* < 0.05) gizzard weights, followed by birds fed with treatment T1, and the lowest was from birds on treatment T2.

Table 7 indicates that, at 24 h post-slaughter, there were significant quadratic trends observed for meat pH_24_ (*y* = 5.79 (±0.019) + 0.011 (±0.006) *x* − 0.001 (±0.0004) x^2^; R^2^ = 0.177, *p* = 0.043) and hue angle_24_ (*y* = 1.38 (±0.022) + 0.016 (±0.007) x − 0.001 (±0.0005) x^2^; R^2^ = 0.180, *p* = 0.041) in response to protease treatment levels. However, no dietary influences were observed on all the meat quality parameters of Cobb 500 broiler chickens.

## 4. Discussion

### 4.1. Growth Performance and Hemato-Biochemical Parameters

This study investigated the effectiveness of pre-treating dietary seaweed with a combination of a proteolytic enzyme and fibrolytic multi-enzymes as a strategy to improve physiological parameters and meat quality of broiler chickens. Repeated measures analysis revealed a significant diet and week interaction effect on feed intake only, which indicates that the ranking of dietary treatments in terms of feed intake changed as the birds grew older. The results revealed that pre-treatment of green seaweed powder with the exogenous enzymes did not improve growth performance of the birds. Matshogo et al. [12] found that the inclusion of untreated green seaweed at 35 g/kg in Cobb 500 broiler diets compromised the performance and feed efficiency of the birds. This was reportedly due to high levels of indigestible non-starch polysaccharides such as cellulose, hemicellulose, xylans, and ulvans in the seaweed [8]. Moreover, high levels of condensed tannins in green seaweeds could reduce the digestibility of protein by forming tannin-protein complexes that are indigestible by endogenous digestive enzymes [24]. Indeed, the use of untreated green seaweed (*Ulva lactuca*) at a rate of 30 g/kg in broiler diets did not improve body weight gain or feed conversion ratio of the birds [25]. It was, therefore, expected that pre-treatment of the seaweed with both the protease mono-enzyme and cellulolytic multi-enzymes would improve the utilization of the diets resulting in improved digestibility and growth performance. The lack of improvement in the feed value of substrates treated with exogenous fibrolytic and protease enzymes has similarly been reported by Mnisi et al. [26] and Mnisi and Mlambo [27] in Japanese quail fed canola-containing diets. Likewise, Sayyazadeh et al. [28] reported no significant effects on body weight and feed efficiency of chickens fed cereal-based diets supplemented with exogenous enzymes. In contrast, several studies have reported positive results when exogenous enzymes were used in poultry diets [29]. Indeed, feed intake could be fully compensated by the effect of enzyme treatment on feed efficiency so that the birds can meet their nutritional requirement by consuming a smaller amount of feed. The lack of improvement upon pre-treatment of seaweed could be due to other factors (pH, viscosity, etc.) in the gastrointestinal tract of the birds that play a crucial role in the effectiveness of enzyme activity on the animal. The benefits of supplementation with enzymes are more evident during the early stages of the life of birds because of different physiological needs throughout the life of the chicken [30]. The combination of amylase, xylanase, and protease has been reported to enhance nutrient digestibility and chicken performance [31]. These inconsistent reports could be attributed to the various types of feed substrates used in these studies as well as different application methods, enzyme activities, and treatment levels [26]. The lack of differences between the control treatment group (T1) and treatment group (T4) on feed intake of six-week-old broilers further confirms the inefficacy of the enzymes to improve the utility of the seaweeds.

Hematological profiles have been used as indicators of dietary responses in farm animals [32]. The results from this study revealed that feeding enzyme-treated seaweed had no impact on the hematological parameters of the birds, and all the observed values were within the normal range reported for healthy chickens [12,14]. The lack of adverse effects indicates that the dietary treatments did not compromise the health of broiler chickens. Additionally, serum biochemical indices give useful information on the health and nutritional status of animals consuming non-conventional feed ingredients [32,33]. The findings from this study showed that seaweeds treated with exogenous enzyme had no negative effects on the blood parameters of broiler chickens. However, the observed quadratic responses of calcium, phosphorus, and symmetric dimethylarginine to protease treatment levels in this study might be attributed high levels of fiber, sterols, and other bioactive compounds in seaweeds [34]. According to Slominski [16] and Shalash et al. [35] supplementing broiler diets with a combination of xylanase, amylase glucanase, and/or protease did not improve the performance of the birds. The response to a multi-enzyme pre-treatment of substrate depends on various factors, such as age of the birds, genetic strain, chemical composition of the diet, and enzyme dose [36].

### 4.2. Carcass Characteristics, Visceral Organs and Meat Quality Attributes

The effect of supplementing green seaweed with exogenous enzymes on the carcass characteristics, visceral organs, and meat quality of broiler chickens is less reported. From this study, combining proteolytic and fibrolytic enzymes did not result in an improvement in carcass traits and visceral organs. Our findings corroborate Mohammed et al. [37], who observed no effect of the exogenous enzyme supplementation on the carcass weight, abdominal fat, and breast meat weight of broiler chickens. Previous reports on the effects of exogenous enzyme treatments on meat quality and organ weight in broilers have been inconsistent. While Symeon et al. [38] reported improvements in meat quality when exogenous enzymes were used, Zakaria et al. [15] did not observe such improvements. An underdeveloped gizzard restricts the broiler chicken’s ability to efficiently digest large feed particles [39]. In the current study, dietary treatments had significant effects on gizzard weights with heavier gizzards in birds reared on seaweed that was treated with both the fibrolytic and protease enzymes compared to untreated seaweed. This finding was not expected because diets rich in fiber have been reported to cause an increase in gizzard size. The consumption of fibrous diets is expected to induce changes in visceral organ sizes as an adaptation mechanism [40]. However, the relative weights of livers, spleen, proventriculus, and intestines were not affected by the diets.

The current study showed that feeding seaweed treated with fibrolytic and protease enzymes had no effect on the carcass characteristics of broiler chickens. Similarly, Fischer et al. [41] found that a multi-enzyme complex did not improve nutrient digestibility and growth performance in broiler chickens. The color of the meat is the most important indicator when consumers buy meat products and is the major factor that affects consumer acceptance of the meat [42]. In this study, no dietary influences were observed on color indicators, which shows that enzyme-treatment of seaweed had no influence on the appearance of the meat. Meat pH is influenced by glycogen levels in meat muscle before slaughter and the extent to which glycogen is converted to lactic acid after slaughter [43]; this explains the drop in meat pH values measured 24 h post-slaughter. Likewise, Hossain et al. [44] reported that meat pH is a direct indicator of the consistency of the muscle acid content. It is not clear why meat pH and hue angle measured 24 h post-mortem quadratically responded to protease enzyme levels; this signifies a need for more research to understand the effect of exogenous enzymes on meat quality attributes. Nonetheless, the observed pH values were in line with the normal pH values reported for broiler meat [45]. Pre-treating seaweed with the combination of fibrolytic and proteolytic enzymes did not improve feed intake, physiological parameters, or meat quality characteristics, which suggests that other feed additives should be evaluated to improve the feed value of dietary seaweed in broiler chicken diets.

## 5. Conclusions

Pre-treatment of dietary green seaweed powder with a combination of a protease mono-enzyme and fibrolytic multi-enzymes did not improve feed intake, physiological, or meat quality parameters in Cobb 500 broiler chickens. In addition, an optimum protease treatment level could not be determined using the growth performance data, indicating a need to investigate levels beyond 15 g/kg of the protease mono-enzyme to generate non-linear responses. Future studies should be designed to investigate the effect of seaweed extracts, instead of the meal, on nutrient digestibility, growth performance, and meat quality parameters of broiler chickens.

## Figures and Tables

**Table 1:** Ingredient composition (g/kg as fed basis) of the experimental diets.

	Grower (14–28 d)					Finisher (29–42 d)				
Ingredients	T1	T2	T3	T4	T5	T1	T2	T3	T4	T5
Viscozyme^®^ L	0	12.0	12.0	12.0	12.0	0	12.0	12.0	12.0	12.0
Protease	0	0	5.0	10.0	15.0	0	0	5.0	10.0	15.0
Seaweed	35.0	35.0	35.0	35.0	35.0	35.0	35.0	35.0	35.0	35.0
Yellow maize	648.8	648.8	648.8	648.8	648.8	646.3	646.3	646.3	646.3	646.3
Extruded full fat soya	31.88	31.88	31.88	31.88	31.88	34.57	34.57	34.57	34.57	34.57
Soya O/C 47%	211.9	211.9	211.9	211.9	211.9	218.1	218.1	218.1	218.1	218.1
Sunflower O/C 36%	41.51	41.51	41.51	41.51	41.51	30.00	30.00	30.00	30.00	30.00
Limestone	7.89	7.89	7.89	7.89	7.89	7.05	7.05	7.05	7.05	7.05
Monocalcium phosphate	8.28	8.28	8.28	8.28	8.28	5.99	5.99	5.99	5.99	5.99
Sodium bicarbonate	1.5	1.5	1.5	1.5	1.5	1.5	1.5	1.5	1.5	1.5
DL-Methionine	2.92	2.92	2.92	2.92	2.92	2.29	2.29	2.29	2.29	2.29
L-Threonine	0.98	0.98	0.98	0.98	0.98	0.58	0.58	0.58	0.58	0.58
Lysine HCL	3.40	3.40	3.40	3.40	3.40	1.85	1.85	1.85	1.85	1.85
Crude soya oil mixer	-	-	-	-	-	11.4	11.4	11.4	11.4	11.4
Lignobond	2.5	2.5	2.5	2.5	2.5	2.5	2.5	2.5	2.5	2.5
Premix	2.5	2.5	2.5	2.5	2.5	2.0	2.0	2.0	2.0	2.0
AxtraPhy10000 Broiler	0.1	0.1	0.1	0.1	0.1	0.1	0.1	0.1	0.1	0.1
Salinomycin 12%	0.5	0.5	0.5	0.5	0.5	0.5	0.5	0.5	0.5	0.5
Zinc Bacitracin	0.33	0.33	0.33	0.33	0.33	0.33	0.33	0.33	0.33	0.33

T1 = standard grower or finisher diet containing 35 g/kg untreated seaweed; T2 = standard grower or finisher diet containing 35 g/kg seaweed pre-treated with 12 g/kg fibrolytic enzymes; T3 = standard grower or finisher diet containing 35 g/kg seaweed pre-treated with 12 g/kg fibrolytic enzymes and 5 g/kg protease mono-enzyme; T4 = standard grower or finisher diet containing 35 g/kg seaweed pre-treated with 12 g/kg fibrolytic enzymes and 10 g/kg protease mono-enzyme; T5 = standard grower or finisher diet containing 35 g/kg seaweed pre-treated with 12 g/kg fibrolytic enzymes and 15 g/kg protease mono-enzyme.

**Table 2:** Nutritional composition of the grower and finisher diets containing seaweed powder.

	Grower (14–28 d)	Finisher (29–42 d)
Dry matter	881.6	882.3
Crude protein	192.1	189.5
Metabolisable energy (MJ/kg)	12.92	13.26
AP Lysine	10.65	9.50
AP Methionine	5.60	4.98
AP Threonine	6.90	6.50
Crude fat	35.54	46.87
Crude fibre	51.87	49.88
Ash	35.44	35.16
Available phosphorus	4.20	3.80
Calcium	8.40	7.60
Chloride	3.54	3.29
Sodium	2.16	2.16
Total phosphorus	5.47	4.94

**Table 3:** Effect of diets containing seaweed pre-treated with fibrolytic and protease enzymes on average weekly feed intake (g/bird) in Cobb 500 chickens.

	^1^Diets						*p* Value	
	T1	T2	T3	T4	T5	^2^SEM	Linear	Quadratic
Week 3	513.4	499.0	475.3	480.8	494.2	14.58	0.892	0.207
Week 4	852.9	809.5	754.3	770.2	774.2	28.83	0.528	0.355
Week 5	1062.0	985.7	989.1	983.4	1015.6	27.86	0.514	0.616
Week 6	1144.5 ^b^	1006.6 ^a^	989.9 ^a^	1031.6 ^ab^	988.1 ^a^	29.60	0.916	0.645

^a,b^ In a row, means with similar superscripts do not differ (*p* > 0.05). ^1^Diets: T1 = standard grower or finisher diet containing 35 g/kg untreated seaweed; T2 = standard grower or finisher diet containing 35 g/kg seaweed pre-treated with 12 g/kg fibrolytic enzymes; T3 = standard grower or finisher diet containing 35 g/kg seaweed pre-treated with 12 g/kg fibrolytic enzymes and 5 g/kg protease mono-enzyme; T4 = standard grower or finisher diet containing 35 g/kg seaweed pre-treated with 12 g/kg fibrolytic enzymes and 10 g/kg protease mono-enzyme; T5 = standard grower or finisher diet containing 35 g/kg seaweed pre-treated with 12 g/kg fibrolytic enzymes and 15 g/kg protease mono-enzyme. ^2^SEM = standard error of the mean.

**Table 4:** Overall body weight gain, overall feed conversion efficiency, and final body weight of Cobb 500 chickens fed diets containing seaweed pre-treated with fibrolytic and protease enzymes.

	^1^Diets						*p* Value	
	TI	T2	T3	T4	T5	^4^SEM	Linear	Quadratic
^2^Overall BWG (g/bird)	1708.0	1658.6	1646.1	1645.3	1653.6	40.00	0.926	0.782
^3^Overall FCE	0.478	0.503	0.513	0.505	0.505	0.007	0.951	0.501
Final body weight (g/bird)	1951.3	1896.4	1884.4	1885.1	1893.6	38.82	0.966	0.792

^1^Diets: T1 = standard grower or finisher diet containing 35 g/kg untreated seaweed; T2 = standard grower or finisher diet containing 35 g/kg seaweed pre-treated with 12 g/kg fibrolytic enzymes; T3 = standard grower or finisher diet containing 35 g/kg seaweed pre-treated with 12 g/kg fibrolytic enzymes and 5 g/kg protease mono-enzyme; T4 = standard grower or finisher diet containing 35 g/kg seaweed pre-treated with 12 g/kg fibrolytic enzymes and 10 g/kg protease mono-enzyme; T5 = standard grower or finisher diet containing 35 g/kg seaweed pre-treated with 12 g/kg fibrolytic enzymes and 15 g/kg protease mono-enzyme. ^2^Overall BWG = body weight gain per bird from 2 to 6 weeks of age; ^3^Overall FCE = feed conversion efficiency per bird from 2 to 6 weeks of age. ^4^SEM = standard error of the mean.

**Table 5:** Hemato-biochemical parameters of Cobb 500 chickens fed diets containing seaweed pre-treated with fibrolytic and protease enzymes.

	^1^Diets						*p* Value	
^2^Parameters	T1	T2	T3	T4	T5	^3^SEM	Linear	Quadratic
Hematology								
Hematocrits (%)	34.16	34.41	33.58	36.33	35.91	1.057	0.551	0.061
White blood cells (×10^9^/L)	13.08	12.61	11.66	13.36	12.0	1.501	0.492	0.681
Heterophils (×10^9^/L)	11.36	11.25	11.10	11.31	9.753	1.321	0.715	0.605
Platelets (×10^9^/L)	35.75	41.98	41.36	40.09	32.33	4.460	0.632	0.230
Monocytes (×10^9^/L)	0.620	0.463	0.486	0.660	0.388	0.119	0.716	0.692
Lymphocytes (×10^9^/L)	1.185	1.611	2.136	1.271	1.555	0.362	0.178	0.137
Eosinophils (×10^9^/L)	0.068	0.008	0.008	0.015	0.008	0.029	0.885	0.747
Serum biochemistry								
Glucose (mmol/L)	2.338	2.346	2.416	3.258	2.375	0.246	0.480	0.113
SDMA (µg/dL)	10.08 ^ab^	10.16 ^ab^	16.0 ^b^	11.50 ^ab^	7.50 ^a^	1.494	0.136	0.012
Creatinine (µmol/L)	12.75	12.50	15.33	18.62	15.25	2.354	0.326	0.239
Urea (mmol/L)	16.02	17.34	13.26	19.26	16.50	1.526	0.660	0.711
BUN/CREA	145.4	167.6	121.0	157.4	158.8	33.46	0.941	0.428
Phosphorus (mmol/L)	3.37 ^a^	4.72 ^b^	4.86 ^b^	4.71 ^b^	4.60 ^b^	0.202	0.514	0.486
Calcium (mmol/L)	1.18 ^ab^	1.03 ^a^	1.51 ^b^	1.57 ^b^	1.09 ^ab^	0.134	0.727	0.003
Total protein (g/L)	99.00	103.4	96.66	105.9	92.75	11.44	0.613	0.749
Albumin (g/L)	31.91	37.75	39.91	48.08	34.16	4.946	0.915	0.147
Globulin (g/L)	60.33	45.08	29.41	29.58	49.08	9.738	0.760	0.059
ALB/GLOB	0.658	0.350	5.541	0.733	0.475	2.022	0.697	0.291
ALT (U/L)	164.2	282.5	203.2	223.7	217.3	54.01	0.519	0.548
ALKP (U/L)	33.58	34.58	40.66	36.00	46.75	13.75	0.654	0.883
Total bilirubin (µmol/L)	105.5	161.0	185.2	237.6	165.8	28.89	0.649	0.152

^a,b^ In a row, means with similar superscripts do not differ (*p* > 0.05). ^1^Diets: T1 = standard grower or finisher diet containing 35 g/kg untreated seaweed; T2 = standard grower or finisher diet containing 35 g/kg seaweed pre-treated with 12 g/kg fibrolytic enzymes; T3 = standard grower or finisher diet containing 35 g/kg seaweed pre-treated with 12 g/kg fibrolytic enzymes and 5 g/kg protease mono-enzyme; T4 = standard grower or finisher diet containing 35 g/kg seaweed pre-treated with 12 g/kg fibrolytic enzymes and 10 g/kg protease mono-enzyme; T5 = standard grower or finisher diet containing 35 g/kg seaweed pre-treated with 12 g/kg fibrolytic enzymes and 15 g/kg protease mono-enzyme. ^2^Parameters: SDMA = symmetric dimethylarginine; BUN/CREA = blood urea nitrogen/creatinine ratio; ALB/GLOB = Albumin/Globulin, ALT = Alanine transaminase, and ALK P = Alkaline phosphatase. ^3^SEM = standard error of the mean.

**Table 6:** Carcass characteristics and internal organs (% WCW, unless stated otherwise) of Cobb 500 chickens fed diets containing seaweed pre-treated with fibrolytic and protease enzymes.

	^1^Diets						*p* Value	
^2^Parameters	T1	T2	T3	T4	T5	^3^SEM	Linear	Quadratic
Carcass yield (%)	74.11	73.50	74.18	72.78	73.15	0.710	0.486	0.844
WCW (g)	1445.8	1394.3	1397.5	1372.7	1385.1	32.13	0.726	0.891
CCW (g)	1405.5	1357.8	1355.2	1340.1	1346.8	30.78	0.737	0.884
Wing	5.38	5.60	5.27	5.73	5.70	0.152	0.297	0.364
Breast	23.83	26.90	26.91	27.40	25.73	1.315	0.464	0.365
Drumstick	5.95	6.20	6.05	6.25	6.23	0.115	0.518	0.535
Thigh	7.40	7.67	7.37	7.73	7.42	0.186	0.722	0.968
Liver	1.93	1.81	1.97	1.96	1.91	0.088	0.481	0.246
Gizzard	1.99 ^b^	1.87 ^a^	2.10 ^c^	2.15^c^	2.12 ^c^	0.088	0.022	0.079
Proventriculus	0.46	0.45	0.47	0.46	0.471	0.021	0.552	0.790
Spleen	0.10	0.10	0.11	0.11	0.10	0.006	0.690	0.108
Duodenum	0.65	0.68	0.70	0.72	0.71	0.035	0.545	0.765
Ileum	1.31	1.36	1.29	1.29	1.33	0.055	0.707	0.301
Jejunum	1.35	1.39	1.41	1.40	1.43	0.056	0.693	0.961
Caecum	0.98	1.04	0.96	1.00	1.03	0.072	0.970	0.415

^a,b,c^ In a row, means with similar superscripts do not differ (*p* > 0.05). ^1^Diets: T1 = standard grower or finisher diet containing 35 g/kg untreated seaweed; T2 = standard grower or finisher diet containing 35 g/kg seaweed pre-treated with 12 g/kg fibrolytic enzymes; T3 = standard grower or finisher diet containing 35 g/kg seaweed pre-treated with 12 g/kg fibrolytic enzymes and 5 g/kg protease mono-enzyme; T4 = standard grower or finisher diet containing 35 g/kg seaweed pre-treated with 12 g/kg fibrolytic enzymes and 10 g/kg protease mono-enzyme; T5 = standard grower or finisher diet containing 35 g/kg seaweed pre-treated with 12 g/kg fibrolytic enzymes and 15 g/kg protease mono-enzyme. ^2^Parameters: WCW = warm carcass weight; CCW = cold carcass weight. ^3^SEM = standard error of the mean.

**Table 7:** Breast meat quality parameters of Cobb 500 chickens fed diets containing seaweed pre-treated with fibrolytic and protease enzymes.

	^1^Diets						*p* Value	
^2^Parameters	T1	T2	T3	T4	T5	^3^SEM	Linear	Quadratic
pH_1_	6.23	6.01	6.09	6.04	5.92	0.130	0.689	0.587
L*_1_	44.30	40.06	40.71	36.62	30.74	5.594	0.205	0.558
a*_1_	3.27	3.24	4.18	3.67	4.17	0.460	0.293	0.640
b*_1_	14.40	12.36	15.57	14.59	13.96	1.081	0.390	0.062
Chroma_1_	14.78	12.79	16.19	15.07	14.60	1.055	0.332	0.061
Hue angle_1_	1.35	1.31	1.30	1.32	1.28	0.036	0.617	0.585
pH_24_	5.79	5.80	5.83	5.83	5.77	0.017	0.469	0.043
L*_24_	56.86	57.62	57.64	58.14	54.75	1.863	0.395	0.422
a*_24_	2.56	3.74	2.62	2.53	2.79	0.421	0.173	0.154
b*_24_	18.02	18.56	20.80	18.39	16.88	17.83	0.276	0.223
Chroma_24_	18.20	18.99	20.97	18.58	17.13	20.97	0.248	0.265
Hue angle_24_	1.43	1.37	1.45	1.43	1.40	0.022	0.447	0.041
WBC (%)	86.08	85.01	88.41	88.48	87.69	1.307	0.181	0.125
Drip loss (%)	1.14	1.41	1.82	1.41	1.48	0.378	0.864	0.522
Cooking loss (%)	8.78	7.00	7.43	9.14	8.74	1.306	0.192	0.718
Shear force (N)	1.90	1.92	1.86	1.93	1.90	0.040	0.869	0.766

^1^Diets: T1 = standard grower or finisher diet containing 35 g/kg untreated seaweed; T2 = standard grower or finisher diet containing 35 g/kg seaweed pre-treated with 12 g/kg fibrolytic enzymes; T3 = standard grower or finisher diet containing 35 g/kg seaweed pre-treated with 12 g/kg fibrolytic enzymes and 5 g/kg protease mono-enzyme; T4 = standard grower or finisher diet containing 35 g/kg seaweed pre-treated with 12 g/kg fibrolytic enzymes and 10 g/kg protease mono-enzyme; T5 = standard grower or finisher diet containing 35 g/kg seaweed pre-treated with 12 g/kg fibrolytic enzymes and 15 g/kg protease mono-enzyme. ^2^Parameters: subscript 1 = all parameters were measured 1 h after slaughter; subscript 2 = all parameters were measured 24 h after slaughter; WBC = water-binding capacity. ^3^SEM = standard error of the mean.
